# Supplementary material for: Rational Design of SARS-CoV-2 Spike Glycoproteins To Increase Immunogenicity By T Cell Epitope Engineering
Source: bioRxiv. 2020 Aug 14:2020.08.14.251496. Preprint. [Version 1] doi: 10.1101/2020.08.14.251496 (PMC7430581; doi:10.1101/2020.08.14.251496)
Supplement: 1 [file NIHPP2020.08.14.251496-supplement-1.pdf]

bioRxiv preprint doi: https://doi.org/10.1101/2020.08.14.251496. this version posted August 14, 2020. The copyright holder for this preprint (which was not certified by peer review) is the author/funder. It is made available under a CC-BY 4.0 International license.

Supporting Information

S1 Table. SARS-CoV-2 S protein residues' core, intermediate, and surface definition for EvoDesign.

S2 Table. Seven human coronavirus S proteins.

S3 Table. The full-length sequences of the top ten designs.

S4 Table. The predicted MHC-II T cell promiscuous epitopes of the native SARS-CoV-2 S protein.
